# A Querying Method over RDF-ized Health Level Seven v2.5 Messages Using Life Science Knowledge Resources

**DOI:** 10.2196/medinform.5275

**Published:** 2016-04-05

**Authors:** Yoshimasa Kawazoe, Takeshi Imai, Kazuhiko Ohe

**Affiliations:** ^1^ Department of Healthcare Information Management The University of Tokyo Hospital Tokyo Japan; ^2^ Center for Disease Biology and Integrative Medicine Graduate School of Medicine The University of Tokyo Tokyo Japan; ^3^ Department of Biomedical Informatics Graduate School of Medicine The University of Tokyo Tokyo Japan

**Keywords:** electronic health records, health level seven, information storage and retrieval, Semantic Web, linked open data

## Abstract

**Background:**

Health level seven version 2.5 (HL7 v2.5) is a widespread messaging standard for information exchange between clinical information systems. By applying Semantic Web technologies for handling HL7 v2.5 messages, it is possible to integrate large-scale clinical data with life science knowledge resources.

**Objective:**

Showing feasibility of a querying method over large-scale resource description framework (RDF)-ized HL7 v2.5 messages using publicly available drug databases.

**Methods:**

We developed a method to convert HL7 v2.5 messages into the RDF. We also converted five kinds of drug databases into RDF and provided explicit links between the corresponding items among them. With those linked drug data, we then developed a method for query expansion to search the clinical data using semantic information on drug classes along with four types of temporal patterns. For evaluation purpose, medication orders and laboratory test results for a 3-year period at the University of Tokyo Hospital were used, and the query execution times were measured.

**Results:**

Approximately 650 million RDF triples for medication orders and 790 million RDF triples for laboratory test results were converted. Taking three types of query in use cases for detecting adverse events of drugs as an example, we confirmed these queries were represented in SPARQL Protocol and RDF Query Language (SPARQL) using our methods and comparison with conventional query expressions were performed. The measurement results confirm that the query time is feasible and increases logarithmically or linearly with the amount of data and without diverging.

**Conclusions:**

The proposed methods enabled query expressions that separate knowledge resources and clinical data, thereby suggesting the feasibility for improving the usability of clinical data by enhancing the knowledge resources. We also demonstrate that when HL7 v2.5 messages are automatically converted into RDF, searches are still possible through SPARQL without modifying the structure. As such, the proposed method benefits not only our hospitals, but also numerous hospitals that handle HL7 v2.5 messages. Our approach highlights a potential of large-scale data federation techniques to retrieve clinical information, which could be applied as applications of clinical intelligence to improve clinical practices, such as adverse drug event monitoring and cohort selection for a clinical study as well as discovering new knowledge from clinical information.

## Introduction

### Clinical Data Searches Through Knowledge Level Queries

While secondary use of electronic medical records (EMRs) are widely expected [1,2], medical data in general do not contain adequate amounts of information or knowledge in their original format, making it difficult to retrieve the desired data based on the knowledge in the clinical domain. For example, when we try to screen patients with medication history of "renin angiotensin inhibitors" as possible candidates for a clinical study, it is common for us to prepare a list of drug codes for such drug classes and query a database with the prepared list. If such a query is performed simply using an expression such as "drugs classified as renin angiotensin inhibitors," it will facilitate our use of the database. As a similar example, when we try to screen patients with medication history of "drugs that cause leucopenia," rather than having to list in a query hundreds of codes for drugs showing the adverse events, if drugs that cause leukopenia are identified using external knowledge resources, and if a search is performed over medication data based on the identified drugs, it would facilitate the research use of EMRs.

### Clinical Data Searches Using Life Science Knowledge Resources

The Linked Open Data project [3] is an attempt to facilitate data usage via the Internet by making data available in a standard format based on the resource description framework (RDF). In the field of life science, attempts are being made to further increase the value of data sets by linking and integrating them as Linked Data. The Bio2RDF project [4] aims at linking and using over 20 types of data sets including the Kyoto Encyclopedia of Genes and Genomes (KEGG) [5,6], the Open Biological and Biomedical Ontologies [7], the Universal Protein Resource [8], and the Gene Ontology [9]. In addition, the National Bioscience Database Center and the Database Center for Life Science in Japan act as primary driving forces and conduct various activities to promote the use of life science data resources and abroad as Linked Data [10,11].

Applying RDF to build clinical databases for secondary use facilitates integration of external knowledge resources expressed in RDF. Teodoro et al. [12] developed a Web-based antimicrobial resistance monitoring system that uses a Semantic Web-based approach to promote the integration of heterogeneous data sources. Assélé et al. [13] developed a framework to perform SPARQL Protocol and RDF Query Language (SPARQL) queries on clinical databases to obtain results about antibiotic resistance and compared their approach with existing business intelligence approaches in terms of usability and functionality. Riazanov et al. [14] developed an ontology for the clinical domain and reported that SPARQL queries can be expressed and executed in an ad hoc manner by mapping the developed clinical domain ontology and clinical data. Pathak et al. [15,16] used publicly available life science data resources as Linked Data and searched over EMR databases integrated with these resources through SPARQL federation queries. The above studies attempt to improve search usability and functionality by applying Semantic Web technologies to supplement information lacking in the clinical data with knowledge from external resources. However, these studies dealt with only institution-specific EMR databases, and it is not easy to apply their methods at other hospitals because schemas of EMR databases generally differ between hospitals; thus, the RDF data structures constructed from these schemas also differ. To avoid these problems and make these technologies widely available, we use health level seven version 2.5 (HL7 v2.5) [17] messages as clinical data. HL7 v2.5 is a messaging standard for information exchange between clinical information systems and the most widely implemented standard for health care in the world. It specifies a number of standards, guidelines, and methodologies by which various clinical information systems can communicate with each other. HL7 messages, although not comprehensive, contain several important types of data for clinical research, such as patient demographics and diagnostic disease.

### RDF for Developing Clinical Databases

Applying RDF in developing clinical databases for secondary use provides the following benefits. First, because the RDF data structure is simple, they can express highly heterogeneous data sets including clinical data, disease concepts, drugs, clinical tests, and genome information using a single data model, making it possible to integrate and handle them in a coherent manner. Second, the inference mechanism supports data sets with hierarchical relationships, such as those containing disease and drug information, through an RDF schema (RDFS) [18] vocabularies. With the relational databases typically used in clinical databases, special measures are required to express the hierarchical structures that exist in data. With RDF, however, this can be accomplished simply by adding the rdfs:subclassOf relationship between the resources. Third, RDF identifies resources through uniform resource identifiers (URIs); therefore, data can be shared via HTTP between different network locations. SPARQL federation query integrates publicly available data sets and allows different network locations to refer to and search over these integrated data sets, maintaining high confidentiality of EMRs. This is expected to be useful when developing clinical databases.

### Aim of the Study

Using RDF as the format for HL7 messages, it is possible to integrate large-scale clinical data and life science knowledge resources. In this study, we implement the following measures to verify this approach. We develop a method for converting HL7 messages into RDF data. Noting that publicly available drug databases constitute useful resources for query expansion in clinical data searches, we show how SPARQL describes adverse drug events (ADEs) and perform searches using such SPARQL expressions. We also examine the search performance and discuss the applicability of the proposed approach to the searches over large-scale data.

## Methods

### RDF and SPARQL

Semantic Web technologies use simple data structures to integrate and use data on a Web-level scale. RDF is the most basic technology for standardizing data expressions, and it consists of a set of URI references (U), a set of blank nodes (B), and a set of literals (L). An RDF triple is a tuple of three elements, that is, a subject (s), a predicate (p), and an object (o), that satisfy s ∈ (U ∪ B), p ∈ U, and o ∈ (U ∪ B ∪ L), respectively. The RDF graph is a directed graph of RDF triples. A data schema in RDF is defined by the vocabulary and semantics of the RDFS. The RDFS is a set of vocabulary and inference rules defined for the vocabulary, and the RDF processor executes these inference rules to derive new RDF triples, which are then added to the RDF graph. For example, rdfs:subclassOf is a vocabulary that defines the class–subclass relationship, and this vocabulary is defined by two rules (ie, a transitive rule and a rule to express a lower class instance being also an upper class instance). Through this inference rule, a search over a lower class and its instances becomes possible by using a higher level abstraction as the search terminology.

SPARQL is an RDF query language. It describes, in the query condition, variables of a pattern to match and their values to use for filtering and extracts the subgraphs that match the given pattern from an entire RDF graph so that the corresponding values of the specified variables are obtained. Filtering of values is performed by using FILTER keywords and by computing a boolean value using the values bound to the variables. Examples of typical functions include a function that performs matching of text strings in their regular expressions and functions that perform logic operations. One beneficial feature of SPARQL is that it can handle multiple RDF graphs as a single graph. SPARQL 1.1 further enhances this feature, making it possible for a single federated query [19] to inquire multiple RDF graphs at different network locations. A federated query expression first designates the SPARQL endpoint with a SERVICE keyword and then describes variables of a pattern to match, similar to a regular SPARQL query, in a clause that follows the endpoint. Consequently, using variables, a federation query can describe a query that can search local or remote RDF graphs.

### SS-MIX2: HL7 Message-Based Clinical Data Storage in Japan

We used HL7 messages stored in the Standardized Structured Medical Record Information Exchange version 2 (SS-MIX2) that has been developed to facilitate secondary use of EMRs as a Ministry project in Japan [20,21]. SS-MIX2 defines the specification of a container for storing EMRs, and the main body of the EMRs is the HL7 v2.5 message. It consists of the standardized storage and the annex storage. The standardized storage contains structured clinical data in the form of an HL7 v2.5 message, such as patient demographics, diagnostic disease, medication orders, laboratory test results, and several kinds of examination orders. The annex storage contains nonstructured clinical data, such as clinical reports, examination reports, and imaging data in arbitrary format. Earlier than the development of the SS-MIX2, standardized terminology for drugs, laboratory tests, procedures, and diagnostic disease has also been developed by the Medical Information System Development Center (MEDIS-DC) [22], and exchange rules for clinical information to be conformed with HL7 have also been developed by the Japanese Association of Healthcare Information System Industry [23]. In 2011, the Ministry of Health, Labor, and Welfare adopted these terminologies and exchange rules as the standard specifications for the health and medical care information field, thereby facilitating the development of standardized medical information systems. Against this background, as of July, 2015, the SS-MIX2 storage has been deployed at 518 hospitals in various regions of Japan [24]. Examples of SS-MIX2 storage applications include (1) an intermediate storage linking multivendor systems and electronic medical record/order entry systems, (2) an intermediate storage for linking regional health care systems, (3) a backup data storage for use in the event of a disaster, and (4) a data source for postmarketing survey of drugs and clinical research.

### Structure of SS-MIX2 Storage and HL7 Message

**Figure 1:**
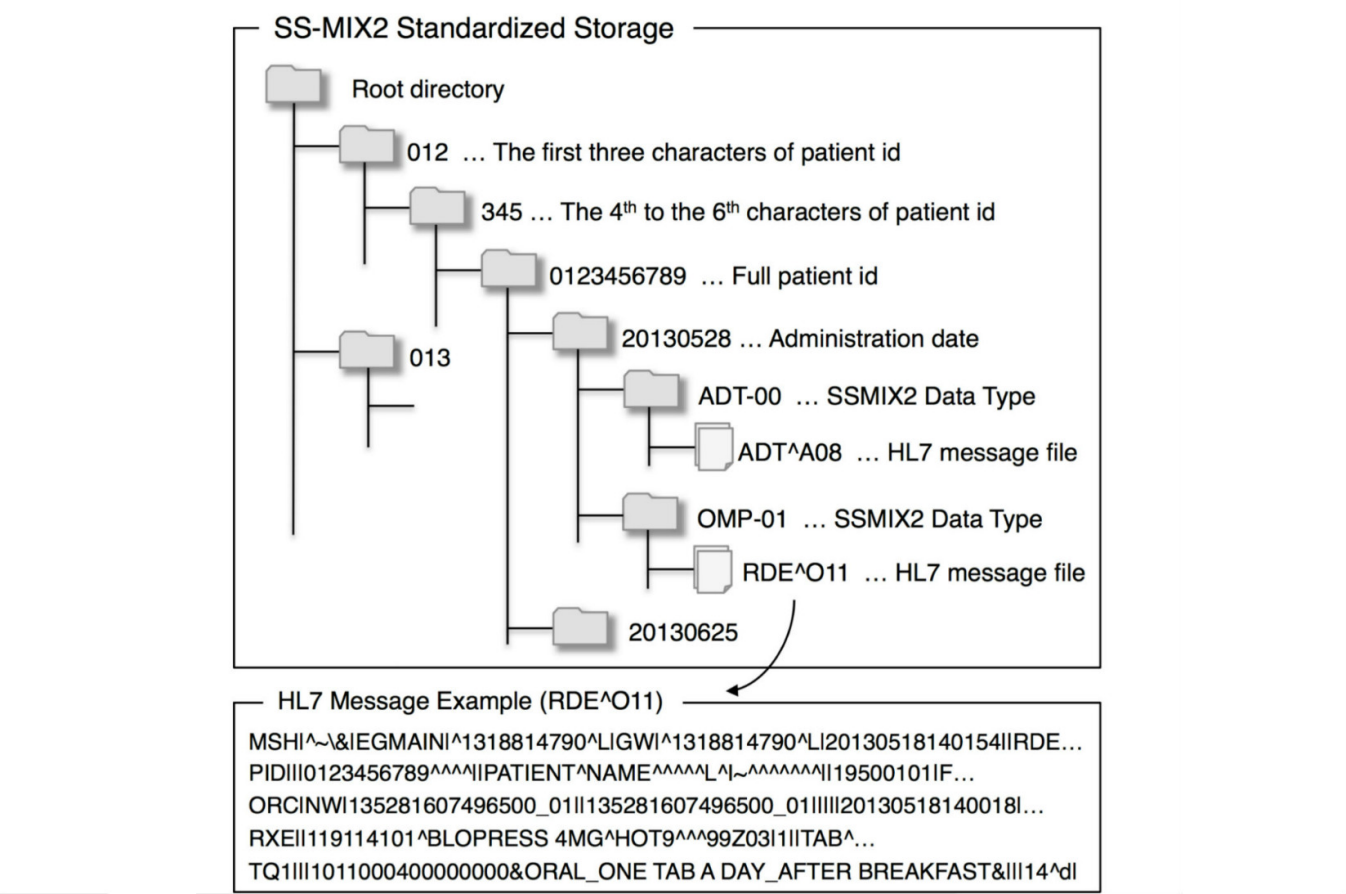
Examples of an SS-MIX2 storage structure and an HL7 message. This example HL7 message (RDE^O11) contains information on a medication order for a patient identified by 0123456789 administered on May 28, 2013. The message contains the following segments: message header (MSH), patient identification (PID), order-related information (ORC), pharmacy encoded (RXE), and timing and quantity (TQ1).

The SS-MIX2 stores HL7 messages below the ordinary directory trees. Under the root directory, patient identifier, administration date, and SS-MIX2 data type are hierarchically located, and corresponding HL7 messages are placed under the bottom directory. The SS-MIX2 data types identify types of clinical information, such as patient demographics, medication orders, and laboratory test results, and these data types are semantically mapped on HL7 message types. For example, HL7 message types to update or delete patient demographics are ADT^A08 and ADT^A23, respectively. SS-MIX2 uses a single data type (ie, ADT-00, for these two HL7 message types). In an HL7 message, each line is called a segment and contains a specific category of information, such as patient identification (PID), order-related information (ORC), and pharmacy (RXE). Each segment consists of a field delimited by a pipe symbol, and the field consists of a field's element delimited by a hat symbol. For example, a patient identifier is located in the third field of the PID segment and a drug code is located in the first field's element in the second field of the RXE segment. Two or more segments may be organized as a logical unit called a segment group, which might or might not repeat. The boundary of the segment group is not identical in a standard form of the HL7 message itself, but it appears in an extensible markup language (XML)-encoded HL7 message described in the next section. Some fields or a field's element may contain a code defined by a certain terminology. In the SS-MIX2, terminologies are used, such as MEDIS DRUG [22] for drugs, JLAC10 [25] for laboratory tests and International Classification of Diseases, and 10th Revision (ICD10) for diagnostic diseases, which are all provided by MEDIS-DC as a nationwide standard. Although these terminologies are unique to Japan except for ICD10, the terminology for drugs can be mapped on the Anatomical Therapeutic Chemical Classification System (ATC) and United States Pharmacopeia (USP) [26] using intermediate resources such as KEGG. This mapping information becomes the key-point to supply an HL7 message with external knowledge recourses by matching a code in the message to a class represented in the recourses. Figure 1 shows examples of an SS-MIX2 storage structure and an HL7 message.

This example HL7 message (RDE^O11) contains information on a medication order for a patient identified by 0123456789 administered on May 28, 2013. The message contains the following segments: message header (MSH), patient identification (PID), order-related information (ORC), pharmacy encoded (RXE), and timing and quantity (TQ1).

### Converting HL7 Messages Into RDF Data

**Figure 2:**
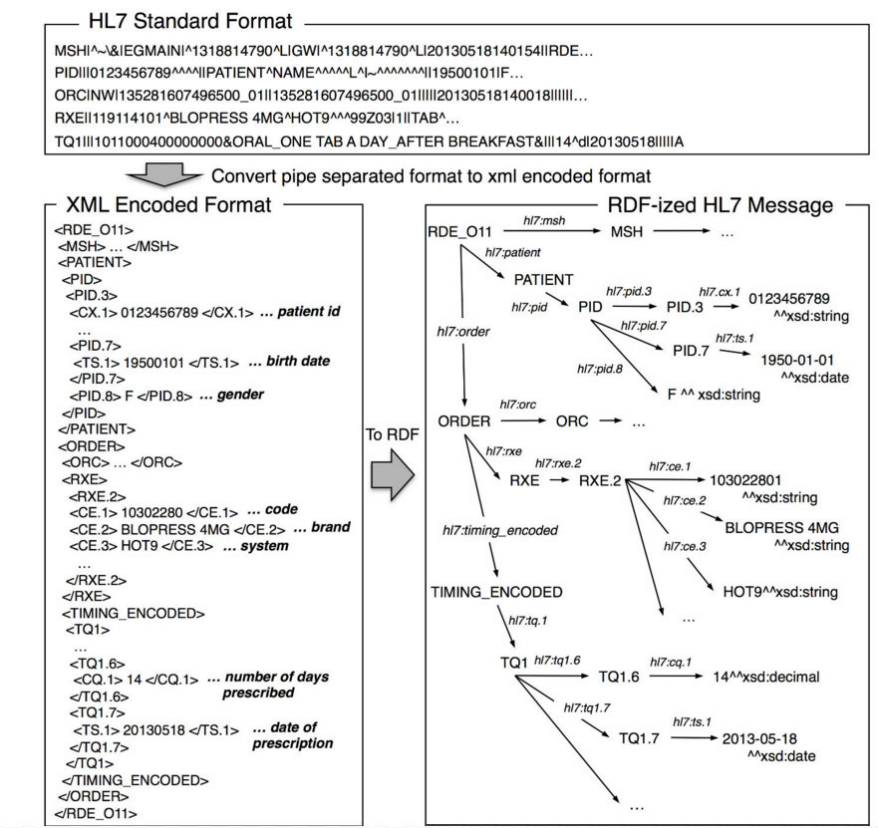
A medication order in the HL7 standard format, XML-encoded format, and after conversion to RDF.

In the standard form of an HL7 message, metadata for fields or a field's elements are not included. For example, the patient’s date of birth is located in the seventh field of the PID segment, although, the message itself does not contain the information. If the name of an RDF resource is determined based on its metadata, HL7 messages are efficiently converted to RDF data. Prasser et al. [27] proposed a method that uses a generic Java-based parser provided by the HL7 Application Programming Interface (HAPI), and that uses the Java class and method names as metadata, traversing Java objects, to convert an HL7 message to RDF data [28]. We also use the HAPI to parse a standard form of the HL7 message, although, we first encode the HL7 message to a form of XML that is also defined in the HL7 specifications. In an XML-encoded HL7 message, segments and segment groups are given in hierarchical XML elements. For example, an XML form of an HL7 message for a medication order starts with an <RDE_O11> tag that describes the type of HL7 message, followed by a tag that describes the segment of a message header <MSH> and segment groups of patient information <PATIENT> and order information <ORDER>. In the segment groups, the corresponding segments are included, such as the PID segment in the PATIENT segment group or the ORC and RXE segments in the ORDER segment group. Similarly, each segment contains a tag for each of its fields to describe either the field or the field's element, such as a time stamp <TS> or a coded character string <CWR>, and text data is marked up with these tags. We then applied a generic method of transformation from XML to RDF [29], in which an RDF resource is generated using the element name of the XML as the name of the resource, creating a subject-predicate-object triple by traversing the hierarchical structure, and mapping the text content to an RDF literal. Note that the mapping needs to be determined in advance because the XML-encoded HL7 message does not contain the data type of the text content. Thus, we sought to map the numerical type of the text content to *xsd:decimal,* the date type to *xsd:date*, the timestamp type to *xsd:dateTime*, and all other types to *xsd:string*. In comparison with the previously mentioned method, there is an advantage to be able to use the names of the segment or field defined by the HL7 specifications, which is not modified depending on the implementation of the Java class and method names. Figure 2 shows a medication order in the standard form of an HL7 message, an XML-encoded HL7 message, and an RDF representation after conversion.

### URI Naming

**Figure 3:**
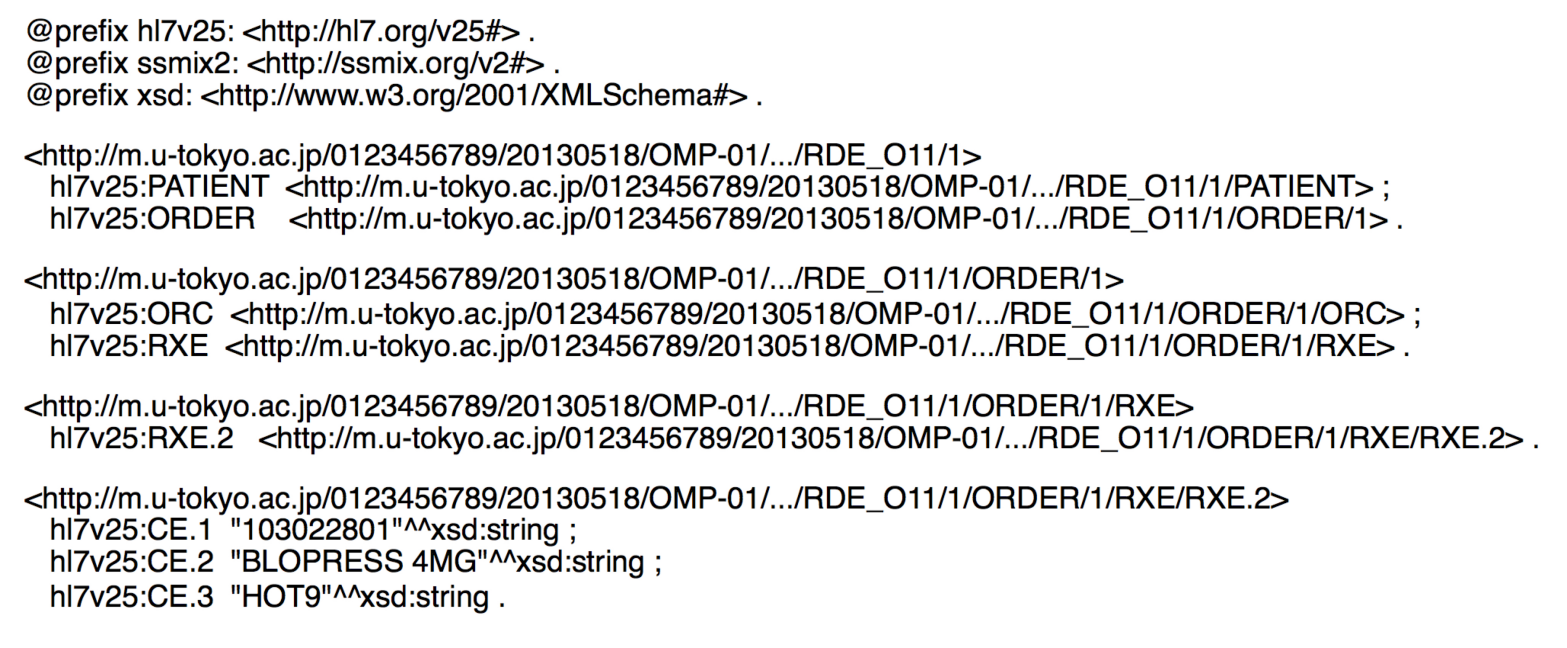
Serialized RDF representation of a medication order in turtle format.

To determine a URI of an RDF resource, we considered two requirements: (1) the name of the URI should preferably contain a structured path to facilitate the application’s access to RDF resources [30], (2) the name of the URI should be generated uniquely from the available information for an HL7 message to avoid redundancy of referring to an RDF repository each time when determining it. To satisfy these requirements, we constructed the name of the URI by connecting a directory path to an HL7 message file, which is already unique in SS-MIX2 storage, with a path to an element in XML that is encoded from the HL7 message. Note that as several HL7 segment groups, such as ORDER and RESULT may appear multiple times in the same hierarchy layer in the XML, duplication of the path names should be avoided by counting how many times they appear in the path. As the HL7 message specifications define which segment groups may appear multiple times, the name of the URI can uniquely identify the deepest elements by considering the duplication. This naming method depends on SS-MIX2 in terms of using the directory path to an HL7 message, although, if only the path to an HL7 massage is uniquely determined, any other way can be applied. Figure 3 shows a portion of a serialized RDF representation of a medication order.

Depending on the purpose of use of the HL7 message, it may contain numerous redundant segments, fields and field's elements, and it may not be necessary to convert all content to RDF data. For example, a MSH segment that provides header information for communication between systems, as well as fields other than the patient identifier, date of birth, and gender in a PID segment, is not required in clinical research. Therefore, when converting to RDF, the amount of RDF data to generate is reduced by only using the segments and fields that are needed for the purpose.

### Query Expansion Using Linked Drug Data

**Figure 4:**
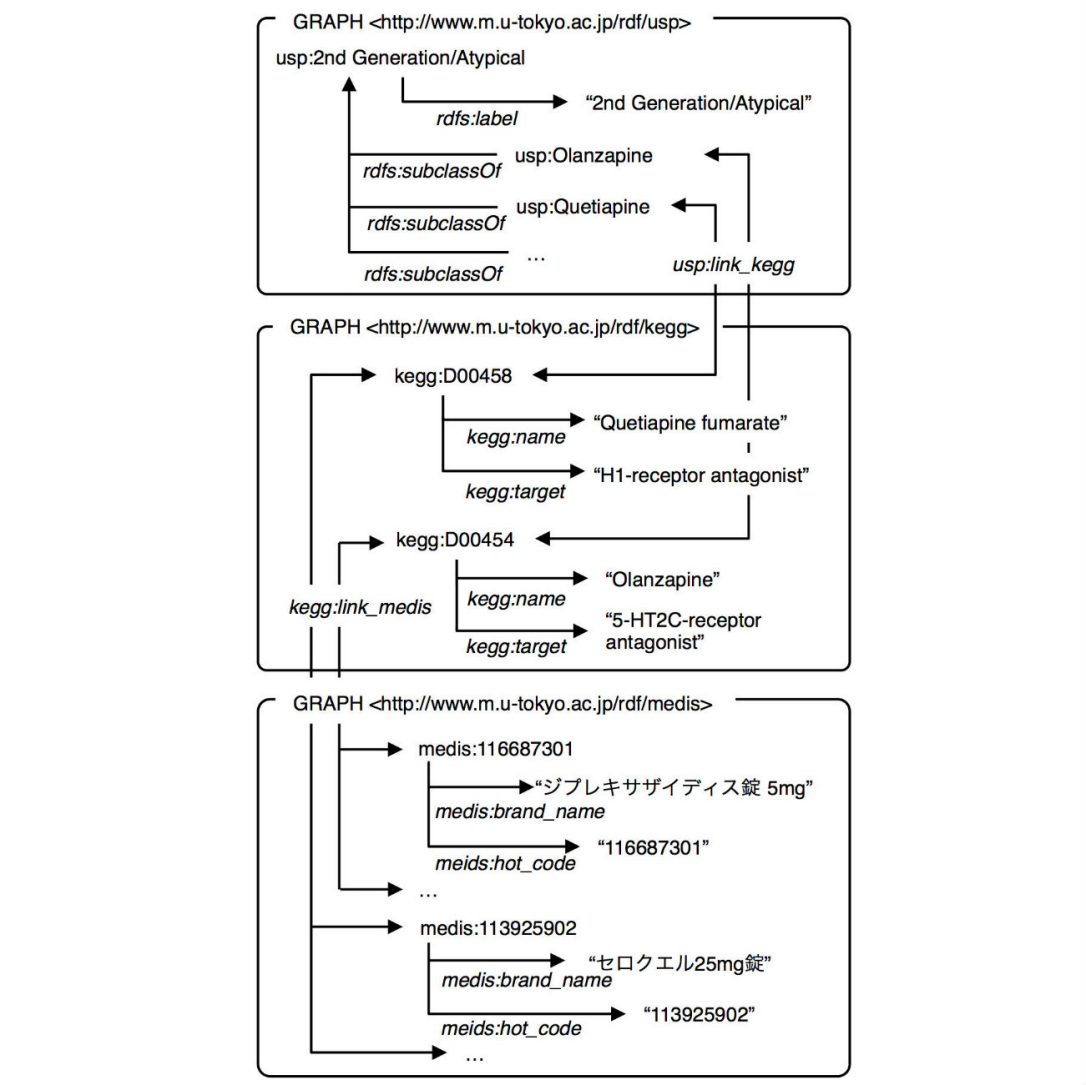
Relationships between USP, KEGG, and MEDIS DRUG used in search for atypical antipsychotic drugs that have an inhibitory effect on the 5HT2C receptor or the H1 receptor.

If a type of drug is identified by its detailed information, it is useful for a query to search for ADEs of a drug. By converting drug databases to Linked Data, it is possible to identify drugs through expressions that use their detailed information and to resolve the identified drugs to their codes used in the HL7 message. For example, a medication order search for atypical antipsychotic drugs that have an inhibitory effect on the serotonin 2C (5HT2C) receptor or the histamine H1 (H1) receptor consists of the following steps: (1) use the USP to identify drugs classified as atypical antipsychotic drugs, (2) use a link between the USP and KEGG to identify corresponding KEGG drugs. Then, narrow down the list to those drugs that have an inhibitory effect on the 5HT2C receptor or the H1 receptor, (3) use a link between the KEGG and MEDIS DRUG to identify corresponding drugs on the MEDIS DRUG and to identify the codes of the drugs to use in the HL7 message, and (4) Use the identified drug codes to search for a medication order over HL7 messages. Figure 4 illustrates relationships between USP, KEGG, and MEDIS DRUG used in this search.

To enable this method, we converted publicly available drug databases into RDF and provided explicit links among the corresponding items to obtain linked data. Because there were no data sources publicly available in RDF format, we converted each source individually to RDF. We got the sources of ATC, USP, and KEGG from a website of the KEGG and made the explicit links based on the information obtained from the KEGG. We used rdfs:subclassOf to describe the higher and lower level relationship in the ATC and USP, and inference was executed and materialized in advance. We also got the sources of SIDER 2 (SIDe Effect Resource) [31] and MEDIS DRUG from each website. In the SIDER 2 dataset, drug classes are coded in STITCH [32] identifiers and names of ADEs are coded in MedDRA along with upper and lower bound of the frequency. The information to link between the SIDER 2 and ATC were obtained from website of STITCH. We used the MEDIS DRUG to match the drug concept in the KEGG to the drug code used in the HL7 message, and the information to link between them were obtained from the the KEGG source. This linked drug data set is hereafter referred to as Linked Drug Data. A summary of the Linked Drug Data is shown in Table 1. The Linked Drug Data is available from our project repository [33].

**Table 1:** A summary of the linked drug data.

Original drug databases	Descriptions	Link to the other databases	Number of drug classes (triples)
Anatomical Therapeutic Chemical Classification System (ATC)	A drug classification system developed by World Health Organization. It divides drugs into different classes according to the organ or system on which they act or their therapeutic and chemical characteristics, such as antihypertensives and the cardiovascular system. In converting to RDF, we used rdfs:subclassOf to represent the hierarchical relationships and added links to the drug classes of KEGG and SIDER 2 at the chemical substance subgroup level.	KEGG, SIDER 2	5770 (48,504)
United States Pharmacopeia Classification (USP)	A drug classification system developed by the US Pharmacopeial Convention. It contains approximately 50 categories, which are typically based on diseases or symptoms that drugs are used to treat, such as pain and psychosis. In the same way as ATC, the hierarchical relationships were represented by rdfs:subclassOf.	KEGG	1459
			(7567)
SIDER 2	A resource that contains ADEs and their frequency, which are extracted from package inserts and publicly available documents. The drugs are coded by STITCH compound identifiers, and the ADEs are described in the preferred terms of MedDra.	ATC	997
			(7,848,862)
KEGG	A resource that consolidates drug data from Japan, the Unites States, and Europe. It organizes drug data based on their chemical structures and ingredients and adds information on their molecular interactions including chemical drug targets and metabolic enzymes. Many entries also include their mapping to other drug databases, and we use the mapping information to establish links to ATC, USP, and MEDIS DRUG.	ATC,	5780
		USP,	(109,976)
		MEDIS DRUG	
MEDIS DRUG	A standard drug terminology that maps various drug terminologies used in Japan. We used MEDIS DRUG to match the drug code in KEGG to the drug code used in the HL7 message.	KEGG	26,126
			(387,319)

### Temporal Patterns to Determine Adverse Drug Events

To identify adverse events, a query condition needs to describe the temporal relationship between the administration of a drug and the adverse events that were assumed to be caused. We classify the temporal relationships into the following four types of basic temporal patterns and explain query expressions using these patterns to identify adverse events.

#### Temporal Pattern 1: Searching for all Medication Orders

This pattern is used to retrieve all medication orders of a specific drug without considering their temporal relationships with other events. This is the most basic pattern of clinical data searches.

#### Temporal Pattern 2: Searching for Adverse Events During Each Medication Period

This pattern estimates the medication period as beginning on the day that a drug medication order was issued and continuing for the number of days prescribed, and it searches for the adverse events during the estimated medication period. Although the medication period estimated in this pattern is likely to be close to the actual drug administration period, irregular medication orders, when issued, could make a period when a drug has been administered appear as it had not been, and the estimated medication period could erroneously exclude such periods. Consequently, it is possible to overlook adverse events during such excluded periods.

#### Temporal Pattern 3: Identifying Adverse Events During a Period Between the Initial and Final Medications

This pattern assumes that the impact of a certain drug extends from its initial medication date to its final medication date, and it is to identify the adverse events during this period. The drug administration period estimated in this pattern could include extended time periods during which the drug had not been administered, and thus, it is possible that the defined drug administration period significantly deviates from the actual drug administration period. However, because the effects of some drugs could continue for an extended time period after drug administration has ended, this pattern identifies adverse events of these types of drugs whose effects extend beyond the end of the medication period.

#### Temporal Pattern 4: Excluding Adverse Events Immediately Before an Initial Medication

This pattern is to increase the degree of certainty of a causal relationship between a drug and an adverse event by excluding the adverse events immediately before initial medication of the drug.

### Experiment Settings

In the next section, we first show a summary of created RDF data to use in this experiment. To ensure the impartiality of the benchmark results, all segments of the HL7 messages were converted to RDF data, rather than arbitrarily deleting unnecessary segments. We then explain three types of query in use cases for detecting ADEs, which are available in our proposed method and show the execution results of searches using these queries. The goal of our experiments was not to investigate specific adverse events, but rather show that it is possible to search over the RDF-ized HL7 messages using SPARQL queries that combine external knowledge and temporal patterns. So, we finally present results of a benchmark that measures the execution time to show that the searches over RDF-ized HL7 messages through SPARQL provide a feasible response speed.

**Figure 5:**
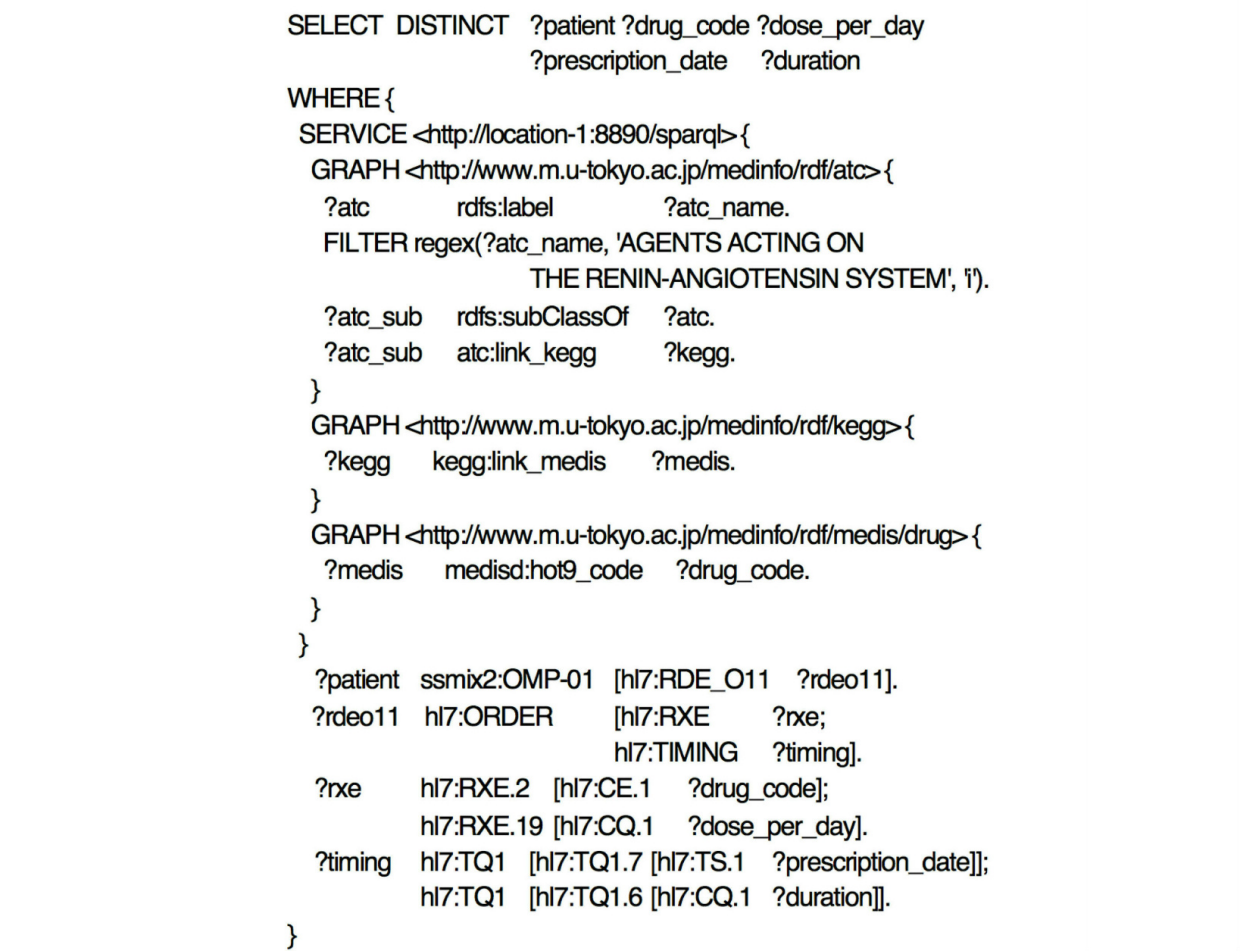
SPARQL expression of Query 1. This query searches all medication orders for drugs classified as renin angiotensin inhibitors.

**Figure 6:**
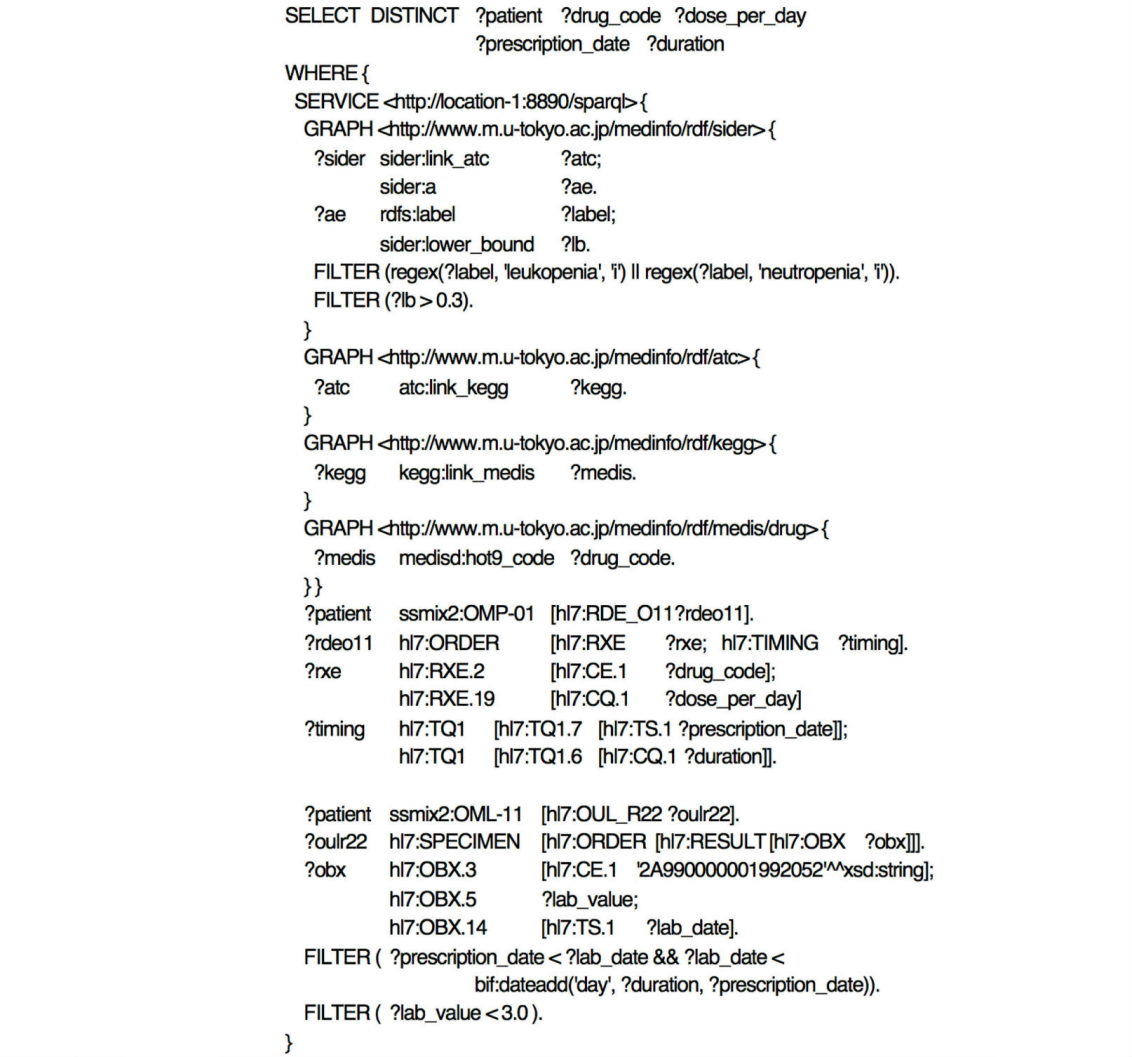
SPARQL expression of Query 2. This query searches all cases for which a leukocyte count of 3000 or less was observed during the medication period of drug types having leukopenia or neutropenia as adverse events.

**Figure 7:**
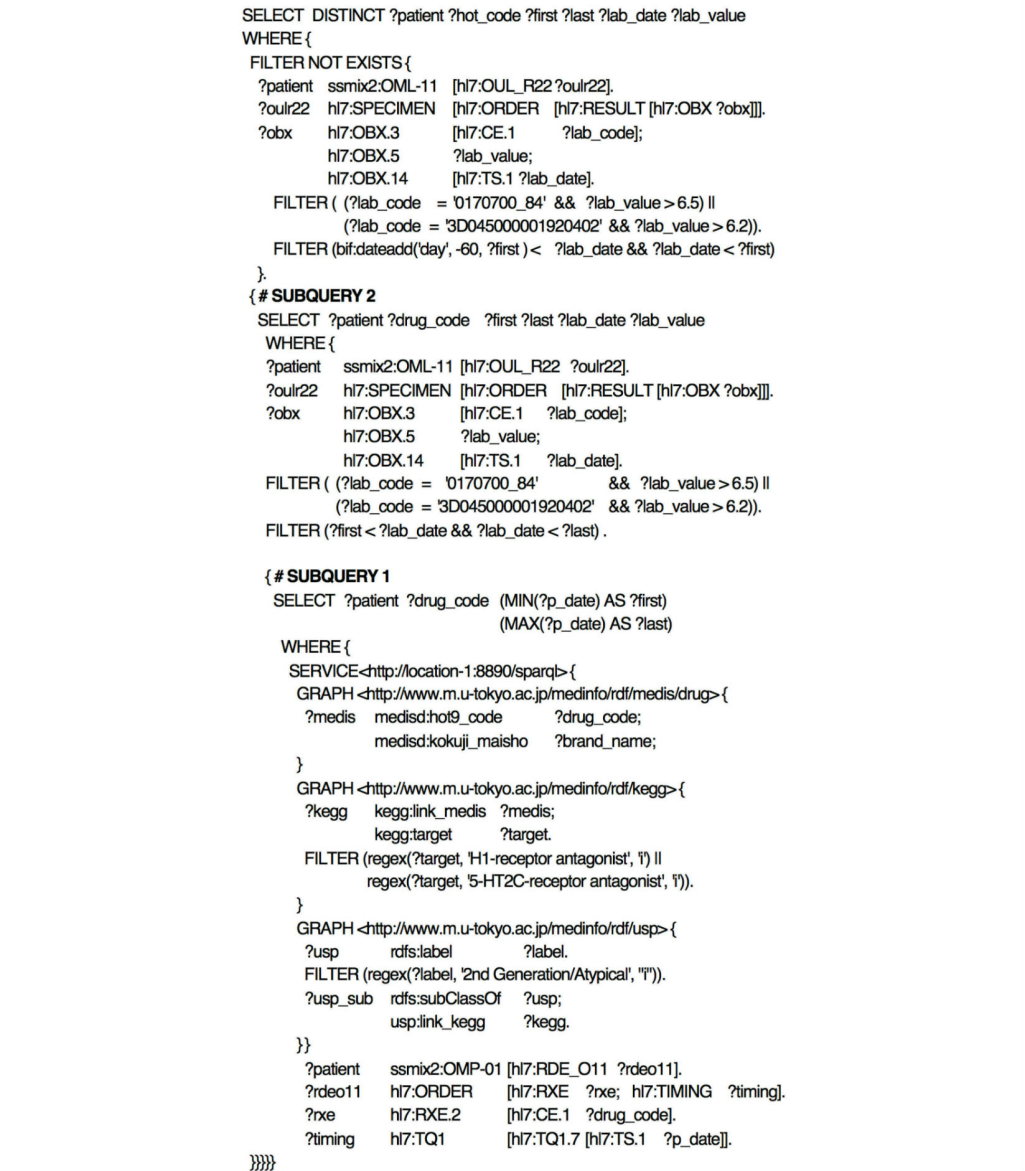
SPARQL expression of Query 3. This query searches all cases satisfying the criteria for impaired glucose tolerance during a period between the initial and final medications of atypical antipsychotic drugs that have a 5HT2C or H1 receptor inhibitory effect. The clinical cases that satisfy the above criteria within 60 days of the initial medication are excluded. In this query, two subqueries are used. In subquery 1, the cases having the period of initial and final medications of the atypical antipsychotic are identified. In subquery 2, the cases satisfying the criteria for impaired glucose tolerance during the period are identified.

To show the relationships between the execution time of the query and the amount of data, we divided whole HL7 messages equally into 10 subdatasets in which the HL7 messages were arranged in ascending order of the date of administration. Then, we measured the execution time of the queries issued five times at each point by increasing every subdatasets. We tested two types of query expressions for each three query in order to compare our proposed query expression with a conventional one. The proposed query uses the Linked Drug Data dynamically by SPARQL federation function in the manner as shown in Figures 5 to 7 below. The conventional query enumerates the individual drug codes in SPARQL filter keyword in advance, which were obtained from the Linked Drug Data separately. Thus, the execution time of the proposed query included, (1) a time to search for individual drug codes from an expression like "renin angiotensin inhibitors" by accessing to Linked Drug Data and (2) a time to search for medication records of RDF-ized HL7 data based on the searched drug codes. On the other hand, the execution time of the conventional query did not include a time to search for the individual drug codes because they are enumerated in advance.

We measured the execution time after relaunching the RDF store and clearing the cache each time a query was executed. Therefore, the execution time included the time it takes to load the data to memory, execute the query, and display the execution results. As we observed that the execution speed dropped drastically when SPARQL queries were not completely optimized through automatic optimization, we manually optimized the execution sequences and then locked them using functionality available in Virtuoso. With regard to the environment for executing queries, the RDF-ized HL7 messages and the Linked Drug Data were stored in two different SPARQL endpoints on a secure network. For the RDF-ized HL7 messages, we used hardware with Intel Xeon 2.60 GHz processors and 256 GB random access memory (RAM). For the Linked Drug Data, we used hardware with Intel Xeon 2.20 GHz processors and 128 GB RAM. Both pieces of hardware ran the CentOS6.5 operating system, and Virtuoso Open-Source Edition 7.1.0 was used as the RDF store.

## Results

### Converted RDF Data

The University of Tokyo Hospital is an educational hospital with more than 1100 beds and 760,000 visits annually. Since 2011, the hospital has been collecting data in the form of HL7 messages in a SS-MIX2 storage. From these collected data, we used the medication orders and laboratory test results during the 3-year period from January 1, 2011 to December 31, 2013. There were approximately 148,000 unique patients, and the number of HL7 messages included was 1.9 million for RDE^O11 (medication orders) and 2.1 million for OUL^R22 (laboratory test results). We then converted them into RDF using the method explained earlier. Approximately 650 million RDF triples for RDE^O11 and 790 million RDF triples for OUL^R22 were converted, and the average number of triples in one message was 360. It was also that the approximate time to convert HL7 messages into RDF were 17 hours and 30 minutes for RDE^O11 and 25 hours 10 minutes for OUL^R22 when we used single CPU (Table 2).

**Table 2:** Summary of the RDF-ized HL7 messages.

Type of HL7 message	Information content	Number of HL7 messages (million)	Number of RDF triples (million)	Triples in a message	Time to convert HL7 messages into RDF
RDE^O11	Medication order	1.9	650	342	17 hours 30 minutes
OUL^R22	Laboratory test result	2.1	790	376	25 hours 10 minutes
Total	-	4.0	1440	360	42 hours 40 minutes

### SPARQL Expressions for Searching Adverse Events

#### Query 1: Identifying Drugs Based on Pharmaceutical Classification and Searching For All Relevant Medication Orders

This is the most basic query searching medication orders that are classified in a certain pharmaceutical category. The query (Figure 5) searches for all medication orders for drugs classified as renin angiotensin inhibitors. The SERVICE clause that follows the WHERE clause queries the Linked Drug Data stored at a SPARQL endpoint, identifies all ATC subclasses of renin angiotensin inhibitors, and resolves their individual drug codes through the KEGG and MEDIS DRUG. When this finishes, triple pattern matching identifies the patients who were prescribed drugs with the code that the SERVICE clause resolved, and binds the dosage amount, medication date, and number of medication days to their corresponding variables of the patients. Query results are returned in a table with the column names described in variables of the SELECT statement. This query does not consider the temporal relationship with other events; thus, it is for Temporal Pattern 1.

#### Query 2: Identifying Drugs Based on Known Adverse Events and Searching for Adverse Events During the Relevant Medication Periods

This query identifies drugs from known adverse events registered in SIDER 2 and searches for clinical cases that may include adverse events resulting from the identified drugs. Specifically, we consider a query (Figure 6) to identify drugs that cause leukopenia or neutropenia as adverse events in SIDER 2 and to search for the clinical cases where the identified drugs were prescribed and a drop in the leukocyte counts was observed during each medication period. Similar to Query 1, the SERVICE clause identifies the drugs that cause leukopenia or neutropenia at a frequency of 30% or higher in SIDER 2 and resolves their individual drug codes through the ATC, KEGG, and MEDIS DRUG. When this finishes, triple pattern matching binds the drug codes of the prescribed drugs, dosage amounts, medication dates, duration of each medication, leukocyte counts, and its examination date to their corresponding variables, and then searches for clinical cases where the leukocyte counts was 3000 or less during the medication period (defined as the period starting on the day of the medication order and continues for the number of prescribed days). Because this query searches for adverse events during each medication period, it is for Temporal Pattern 2.

#### Query 3: Identifying Drugs Based on Pharmaceutical Classification and Their Targets, and Searching for Adverse Events During the Relevant Drug Medication Periods

This query illustrates that when multiple drug data resources are used, drugs can be identified with more detailed characteristics. In clinical backgrounds, atypical antipsychotic drugs are known to have a tendency to trigger diabetes. It is hypothesized that these drugs cause chronic bulimia by blocking 5HT2C and H1 receptors and bring about obesity and hyperinsulinemia, thereby inducing diabetes [34]. This query may help examine this hypothesis through identifying the drugs that demonstrate these characteristics and extracting clinical cases that satisfy the criteria for diabetes during the medication period. As mentioned above, this query (Figure 7) first narrows down drugs classified as atypical antipsychotic drugs in the USP classification to those in KEGG having an inhibitory effect on 5HT2C or H1 receptors, and then resolves individual drug codes through MEDIS DRUG. It then uses a filter operation to derive the initial and final medication dates for each patient from the medication orders of the drugs with the resolved drug codes, and extracts clinical cases where the HbA1c value or the serum glucose satisfies the criteria for impaired glucose tolerance during the medication period. Note that as the HbA1c value changes gradually, we used the period between the initial and final medications, rather than using each medication period. We also added a condition to exclude clinical cases satisfying the same criteria within 60 days of the initial medication. Therefore, this query is for a combined temporal pattern of Temporal Patterns 3 and 4.

### Execution Results of Each Query

Table 3 shows the results of executing each query over the RDF-ized HL7 messages for a 3-year period. The Query 1 expression of "drugs classified as renin angiotensin inhibitors" yielded 476 different types of drug codes by the Linked Drug Data, and there were a total of 197,366 medication orders found for these drugs. Similarly, the Query 2 expression of "drug types having leukopenia as adverse events" yielded 131 types of drug codes using SIDER 2, and the Query 3 expression of "of the atypical antipsychotic drugs, those having a 5HT2C or H1 inhibitory effect" yielded 78 drug types, with Queries 2 and 3 obtaining 1171 and 58 results, respectively.

### Query Execution Performance

**Figure 8:**
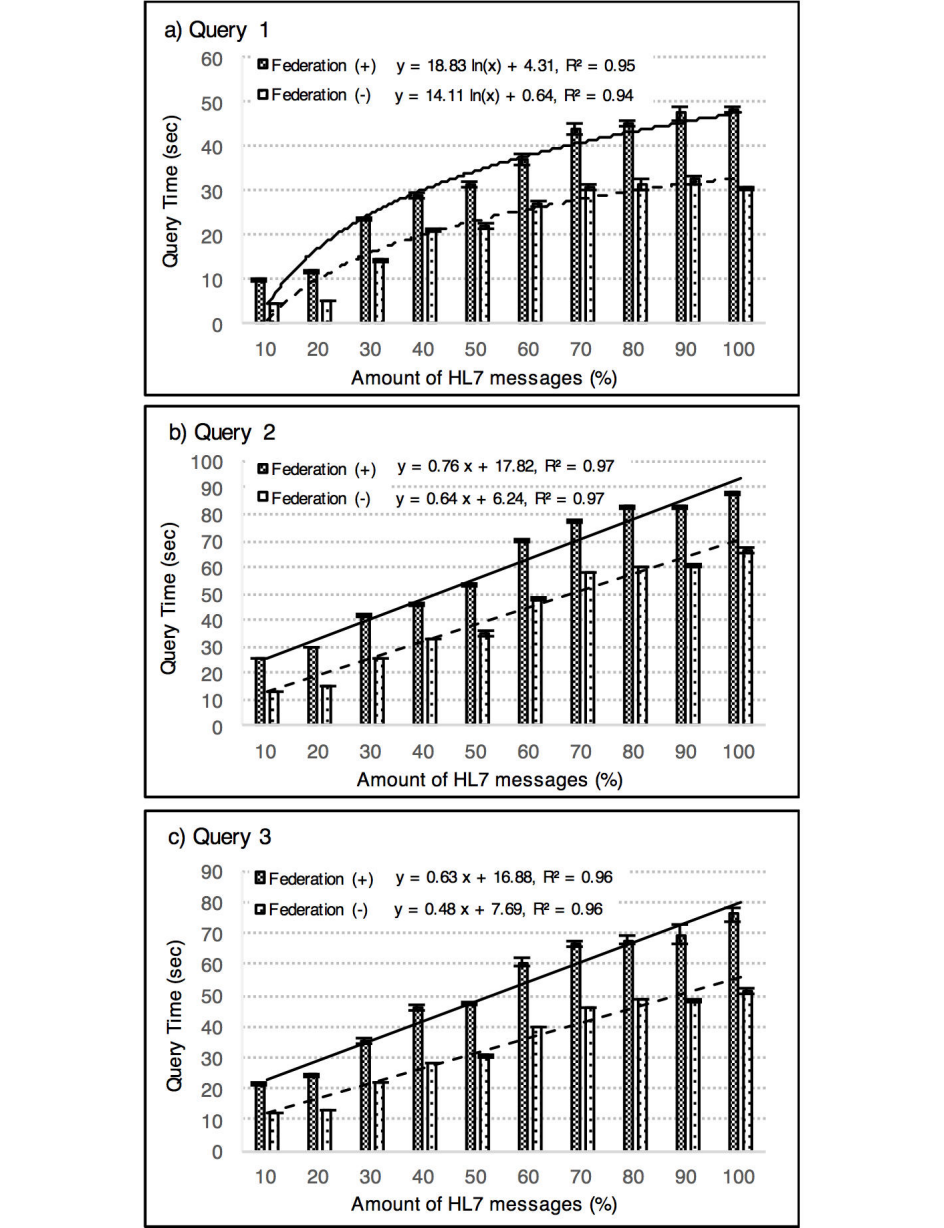
The average measured execution times of Queries 1, 2, and 3 obtained through the experiments are shown in a), b), c), respectively. In each subfigure, bar graphs represent the average measured execution times of the two types of query expression with standard errors, and solid or dashed line represent the approximate average execution times.

Figure 8 shows, for each three query, the average measured execution times of the two types of query expression (ie, our proposed query that use Linked Drug Data dynamically with SPARQL federation function and a conventional query in which the individual drug codes are enumerated in SPARQL filter keyword). The average execution time of the proposed queries were significantly longer than the conventional one, and these were 49% longer in Query 1, 43% in Query 2, and 51% in Query 3, in total. It was also that the execution time of the Query 1 showed logarithmic growth, and the Query 2 and the Query 3 showed linear growth. The coefficient of determination in these regressions ranged from 0.94 to 0.97.

**Table 3:** Summary of each query and the respective execution results.

No.	Summary of query condition	Drug data sources to resolve the expression	No. of resolved drug codes	Results
1	Cases for which drug types classified as renin angiotensin inhibitors were prescribed, and all medications of such drugs.	ATC	476	197,366
		KEGG		
		MEDIS DRUG		
2	Cases for which a leukocyte counts of 3000 or less was observed during the medication period of drug types having leukopenia or neutropenia as adverse events, and all corresponding medications.	SIDER 2	131	1171
		ATC		
		KEGG		
		MEDIS DRUG		
3	Cases satisfying the criteria for impaired glucose tolerance during a period between the initial and final medications of atypical antipsychotic drugs that have a 5HT2C or H1 receptor inhibitory effect. Clinical cases that satisfy the above criteria within 60 days of the initial medication are excluded.	USP	78	58
		KEGG		
		MEDIS DRUG		

## Discussion

### Primary Findings

To further improve the usability of EMRs, EMRs need to be integrated with external data sources that serve as knowledge resources. Currently, clinical specialists provide and interpret the knowledge used in making clinical data inquiries and, in many cases, manually translate the knowledge into codes of terminology and describe them in queries. This not only requires time and increases the number of errors [13,15] but also leads to the possibility of differing interpretations of coding, resulting in incompatibility among query results. Semantic Web technology provides a framework for integrating heterogeneous data sets using RDF and enables the extraction of data from multiple endpoints on a network using queries in a uniform format and standard Web protocols. This makes it possibly not only to integrate heterogeneous knowledge resources but also to share publicly available resources as knowledge sources and to handle highly confidential clinical data without compromising their confidentiality.

We converted drug databases to the Linked Drug Data, used them as the knowledge for query expansions, and searched over the RDF-ized HL7 messages. We showed three queries illustrated by the queries for drugs including renin angiotensin inhibitors, as well as more advanced expressions for drugs that cause leukopenia and also for atypical antipsychotic drugs. We only show three queries, although, we believe that wider ranges of queries are possible by combining four temporal patterns and various search expressions to identify drugs. These query expressions require clinical knowledge, and such knowledge must be supplied from external knowledge sources, as clinical data do not contain such knowledge. Our query expression used knowledge of drugs separate from clinical data that exist at a different endpoint on a network through SPARQL's federation query. This suggests that enhancing knowledge resources would improve the search usability of clinical data and the possibility to search over clinical data on a shared knowledge basis.

The Query 1 example resulted in 476 drug code types for renin angiotensin inhibitors. However, in reality, it is unlikely that one hospital adopts all types of renin angiotensin inhibitors, and only a few types are actually adopted by any one hospital. Because different hospitals may adopt different drug types, the drug codes listed for one hospital may not apply to another. The proposed method dynamically resolves the expression like "renin angiotensin inhibitors" using external knowledge resources, enabling clinical data searches using expressions at a level close to the knowledge without considering specific types of drugs that different hospitals may adopt. This not only improves the usability of query expressions for specialists but also suggests the possibility of reusing queries (ie, using the same query at multiple hospitals) [34,35].

The Query 2 example showed a use case for ADEs, which used SIDER 2 to search drugs that potentially cause leukopenia. As for the database of ADEs itself, there is another publicly available database named ADEpedia 2.0 that use RxNorm codes for medications and SNOMED CT or MedDRA codes for phenotypes related to ADEs [36]. In this database, the relationships between the drugs and ADEs are represented by predicates such as 'contraindicated_drug' for information of contraindications and 'causative_agent_of' for adverse drug effects. Although SIDER 2 and ADEpedia 2.0 is useful to search known relationships between the drugs and ADEs, they are not necessarily enough for a use case to investigate unknown ADEs that may be discovered from EMR. To enable this, we needed to complement them by using the different type of drug database. We showed Query 3 example that make use of the information of drug class and type of receptor, which are enabled by linking USP and KEGG. Although this query shows a limited example, increasing variation of the search expression by using multiple drug database is assumed to be useful for investigating ADEs, and in order to do so, it is primarily important that these databases can be linked each other.

We showed a method for converting RDF data not by selecting arbitrary elements contained in the HL7 message but by using all the elements as they are. The reason why is because it was difficult to specify which elements are necessary for a clinical study in advance. As a trade-off, the SPARQL query we showed may be difficult to describe unless we are familiar with the specifications of the HL7 message. The difficulty of describing this SPARQL query will be summarized in the following three points. First, when describing the pattern matching of SPARQL, nesting up to reaching the necessary elements would be considerably deep. For example, until reaching the drug code, it is necessary to pass through five nodes: RDE_O11, ORDER, RXE, RXE.2, and CE.1. For this reason, the user must be familiar with the structure of the HL7 message. In order to solve this problem, it is conceivable to select the elements that are required for a clinical study from the HL7 message, reconstructing a simpler model of RDF data composed of only its elements. To do this, a guideline for which elements should be converted might be useful, and to make such a guideline, it is desirable that Health Level Seven and some associations related to clinical research discuss and select the required elements necessary for clinical researches in general. Second, the vocabulary that is reusable to represent the RDF resource is not used. Some properties such as "patient ID," "birthdate," and "gender" shown in Figure 2 might be good to associate them with the existing vocabulary that is defined in the ontology such as foaf and vCard. However, the vocabulary corresponding to almost all other HL7 elements, including the drug code, medication dose, unit of the dose, and so on did not exist as far as we know. Therefore, in this study, we gave greater importance to keeping the consistency of the method of converting the HL7 to RDF by using the names of the tags obtained when converting the HL7 to XML as the vocabulary rather than reusing only those few vocabulary. Finally, temporal reasoning is important for investigation for ADEs, although, it might be difficult to write it against our RDF-ized HL7 data with SPARQL. We used filter-based solution in Queries 2 and 3 to compare the date of laboratory test results and the date of the medications in order to be able to consider the causal relationships between them. We also used subquery solution in Query 3 to identify the first and the last time of medications of atypical antipsychotic drugs in order to identify diabetes that occurred or not occurred during time frames based on the two time points. Although we showed these queries as possible as simple, they might be typically verbose and difficult to write. It is conceivable that using Allen’s temporal predicates such as "before," "after," and "during" in the pattern matching of the query [14] is useful to avoid the SPARQL filter-based comparison of the time. In order to do that, an interval-based temporal information should be given to the comparable events and they should be connected according to their relationships when the RDF data are created. It might be also that giving a mark to specific time events such as the first and the last time of medications is useful to identify them without the subquery solution. These methods make the description of the query more concise at the expense of computational complexity at the time of creating RDF data. In this study, we did not apply these methods because we focused on using all elements in HL7 message as they are, it would be worth to consider to make the expression of temporal reasoning concise.

Regarding the query execution time, we tested two types of query expression for each three query to show the difference of the execution time between our proposed query expression and a conventional one. As for the conventional expression, the number of the drug codes enumerated in each query were 476, 131, and 78, respectively, as shown in Table 3. The advantage of the proposed query is that the expression is concise and human readable in comparison to the conventional one, and that allows identification of drugs based on the detailed information rather than the drug codes can be listed. On the other hand, the disadvantages are that it is inferior in execution time, it takes approximately 40% to 50% more time than conventional one. It was also that what kind of drug code will be searched is unknown until the query is run. These comparative aspects indicate a trade-off between simplicity of the query expression and the execution time of the query as well as search reliability. In particular, as it is necessary to separately consider the reliability of the drug code obtained by the Linked Drug Data, this can be noted as one of the limitations of this study.

The result of the experiment also showed that the average execution time of the Query 1 showed logarithmic growth, and the time of the Queries 2 and 3 showed linear growth with the coefficient of determination ranged from 0.94 to 0.97. This indicates that these regressions approximated the query execution time well. These results might be counterintuitive especially in the logarithmic growth in Query 1, although, it was assumed to be possible that the logarithmic growth is consistent with computational complexity of B-Tree indices is O(log n), which are used in the RDF database we used. Although the result will not be generalized because an execution time of a query depends on various settings, such as amount of data, the content of the query, and the kind of the database system, the execution time of these queries increased with the amount of data without diverging in our experiments.

### Limitations

We converted HL7 messages to RDF data automatically without changing the HL7 message structures. This suggests that the proposed method can be applied not only at the University of Tokyo Hospital that has adopted SS-MIX2 storage but also at numerous other hospitals that use HL7 messages. To demonstrate this, future research is required to verify the applicability of the proposed method at multiple hospitals. In addition, we considered adverse events cases in our research, and thus, it was medication orders and laboratory test results that were converted to RDF data. However, HL7 messages contain other types of clinical data such as patient demographics, diagnostic disease, and some kind of order information. When these types of clinical data are converted to RDF data, a wider variety of query expressions are required to search over the converted RDF data, and future research should examine such query expressions. We have not verified the drugs identified through our query expansions, nor verified extracted clinical data against the gold standard, and these are the limitations of the research.

### Conclusions

This study applied Semantic Web technology to use publicly available drug databases as the knowledge for query expansions and demonstrated clinical data searches through SPARQL. The proposed method executed queries with knowledge resources separate from clinical data, suggesting that enhancing knowledge resources would improve the usability of clinical data. This study also converted HL7 messages to RDF data using an automatic way without modifying the HL7 message structures and demonstrated searches over the converted RDF data using SPARQL. This suggests that the proposed method can be applied not only at the University of Tokyo Hospital that has adopted SS-MIX2 storage but also at numerous other hospitals that use HL7 messages. We have not verified the drugs identified through query expansions, nor verified extracted clinical data; such verifications will be performed in future research. Future research also includes applying the proposed method at other hospitals and supporting a wider variety of HL7 messages.

## Abbreviations

5HT2C: serotonin 2C
ADE: adverse drug event
ATC: Anatomical Therapeutic Chemical Classification System
EMR: electronic medical record
H1: histamine antagonist of the H1 receptor
HAPI: Health Level Seven application programming interface
HL7: Health Level Seven
ICD10: International Classification of Diseases, and 10th Revision
KEGG: Kyoto Encyclopedia of Genes and Genomes
MEDIS-DC: Medical Information System Development Center
MSH: message header
ORC: order-related information
PID: patient identification
RAM: random access memory
RDF: Resource Description Framework
RDFS: Resource Description Framework Schema
RXE: pharmacy
SIDER 2: SIDe Effect Resource
SPARQL: SPARQL Protocol and RDF Query Language
SS-MIX2: Standardized Structured Medical Record Information Exchange version 2
TQ1: timing and quantity
URI: uniform resource identifier
USP: United States Pharmacopeial Convention Classification System
XML: Extensible Markup Language.
